# Fabrication and characterization of graphene oxide-based polymer nanocomposite coatings, improved stability and hydrophobicity

**DOI:** 10.1038/s41598-023-35154-z

**Published:** 2023-06-02

**Authors:** Sachin Sharma Ashok Kumar, Nujud Badawi M., Khalid Mujasam Batoo, I. A. Wonnie Ma, K. Ramesh, S. Ramesh, Mohd Asif Shah

**Affiliations:** 1 Centre for Ionics University of Malaya, Department of Physics, Faculty of Science, Universiti Malaya, 50603 Kuala Lumpur, Malaysia; 2 King Abdullah Institute for Nanotechnology, King Saud University, P.O. Box-2455, 11451 Riyadh, Saudi Arabia; 3 Department of Physics/Saveetha School of Engineering, Saveetha University (SIMATS), Chennai, India; 4 College of Business and Economics, Kebri Dehar University, 250, Kebri Dehar, Somali Ethiopia; 5 School of Business, Woxsen University, Kamkole, Sadasivpet, Hyderabad, Telangana 502345 India; 6 Division of Research and Development, Lovely Professional University, Phagwara, 144001 Punjab India

**Keywords:** Materials for energy and catalysis, Nanoscale materials

## Abstract

In this study, acrylic-epoxy-based nanocomposite coatings loaded with different concentrations (0.5–3 wt.%) of graphene oxide (GO) nanoparticles were successfully prepared via the solution intercalation approach. The thermogravimetric analysis (TGA) revealed that the inclusion of GO nanoparticles into the polymer matrix increased the thermal stability of the coatings. The degree of transparency evaluated by the ultraviolet–visible (UV–Vis) spectroscopy showed that the lowest loading rate of GO (0.5 wt.%) had completely blocked the incoming irradiation, thus resulting in zero percent transmittance. Furthermore, the water contact angle (WCA) measurements revealed that the incorporation of GO nanoparticles and PDMS into the polymer matrix had remarkably enhanced the surface hydrophobicity, exhibiting the highest WCA of 87.55º. In addition, the cross-hatch test (CHT) showed that all the hybrid coatings exhibited excellent surface adhesion behaviour, receiving 4B and 5B ratings respectively. Moreover, the field emission scanning electron microscopy (FESEM) micrographs confirmed that the presence of the functional groups on the GO surface facilitated the chemical functionalization process, which led to excellent dispersibility. The GO composition up to 2 wt.% showed excellent dispersion and uniform distribution of the GO nanoparticles within the polymer matrix. Therefore, the unique features of graphene and its derivatives have emerged as a new class of nanofillers/inhibitors for corrosion protection applications.

## Introduction

Corrosion is when a metal deteriorates via charge transfer reactions in an ambient surrounding, thus, resulting in the destruction of the metal surface^1–3^. Globally, corrosion has possessed a great threat to society, harmful to humans and a major industrial issue^4–6^. Moreover, it has been reported that it is impossible to completely prevent corrosion, however, it can only be minimized and retarded^7^. Since majority of the industries are facing the challenges related to corrosion, therefore, significant efforts were devoted to develop several vital steps to protect the materials from corroding. Furthermore, despite having numerous corrosion prevention strategies which are in operation, there is still a huge necessity to further increase the life of the components^8^. For instance, methods namely surface treatment, protective coatings, electrochemical cathodic protection and green corrosion inhibitors have been employed to slow down or completely inhibit the main electrochemical occurrence that led to the degradation of metals^9,10^. Interestingly, in modern industry, organic coatings have been widely adapted to prevent the corrosion of metallic structures. In addition, organic coatings have extraordinary features such as low cost, excellent adhesion onto various substrates, high chemical and thermal stability, high electrical resistance, good dimensional stability, high tensile strength and high crosslinking density respectively. Nevertheless, some drawbacks in terms of the corrosion protection such as poor flexibility and impact resistance, permeability of the corrosive agents (e.g., oxygen, water, chloride ions etc.) to the coating/metal interface and the creation of micropores during the coating preparation has been exhibited by means of the neat epoxy resin coatings. Hence, this resulted in the loss of the coating adhesion which further caused the deterioration of the coated substrate^10–14^.

In the recent years, it has been numerously reported that nanocomposite coatings with hydrophobic attributes and organic–inorganic hybrids have demonstrated significant enhancement in the life of materials that were susceptible to corrosion, which resulted in huge savings. Up to date, in order to increase the lifespan of the materials under extreme environmental conditions, the main goal of the industry was to produce robust oxidation and corrosion resistant coatings. Therefore, compared to traditional coatings, the nanostructured materials engineering has enabled a promising pathway to design environmentally friendly anti-corrosive coatings which shown the capability to last much longer^8^. Discovered in 2005, a two-dimensional (2D) material, graphene that was made-up of one atom thick ${sp}^{2}$ hybridized carbon nanostructure had inspired the world and widen the field of application for composite materials^15–18^. Moreover, its distinguished characteristics such as high specific surface area, thermal and chemical stability, chemical inertness, impermeability to ion diffusion, excellent electrical conductivity, and high mechanical strength make this material a promising candidate for corrosion control and protection on metal^19–21^. Nevertheless, the practical use of graphene has been limited due to the difficulty in immobilizing graphene directly on metal surface, poor dispersibility in either aqueous or non-aqueous solvents, costs generated by the fabrication methods and its tendency to agglomerate when used in higher concentrations respectively^22^. In addition, graphene sheets are chemically inert, thus, this resulted in the prevention of various interaction with the polymer matrices, hence, causing extended filler-filler aggregation in composites.

As a result, GO has emerged as a stronger alternative because its structural strength was similar to graphite. Moreover, the GO structure is made up of a hexagonal carbon network with both ${sp}^{2}$ and ${sp}^{3}$ hybridized carbon atoms with epoxide and hydroxyl functional group present on its basal plane and carboxyl and carbonyl groups on the edges respectively^23,24^. Furthermore, the hydrophilic nature of the GO was attributed due to the presence of the oxygen bearing functional groups, thus, resulting the GO to be dispersible in water and polymers. However, through Van der Waals interactions, the GO sheets had the tendency to agglomerate within a polymer matrix. Previously, it has been reported that the coating performance was very much dependent on the size of the graphene nanoparticles^25,26^. Therefore, it is essential to ensure that the functionalization of graphene was performed appropriately to achieve optimum properties and to avoid aggregation of the nanoparticles, hence, existing in a well exfoliated manner in the matrix^27^. For instance, to effectively enhance the properties of the polymeric matrix, it is vital to ensure that the GO dispersed well and uniformly distributed in the polymer coating. However, due to the interaction through the hydrogen bonding between the functional groups that existed on the basal plane of the GO sheets, this has resulted in the difficulty to incorporate GO nanoparticles as a nanofiller in polymer matrices. Different methods are used to overcome this issue, for instance, method, Cano et al. have used vacuum filtration method for the functionalization of GO and polyvinyl alcohol (PVA) to fabricate paper-like composite materials^28^. Here, it was concluded that the incorporation of GO as a nanofiller remarkably enhanced the strength of the nanocomposite coatings. Furthermore, Sachin SA Kumar et al. reported a new method to prepare GO thin films (GOTFs)^29,30^. Here, the GOTF exhibited a high resistance on UV degradation for a duration of 25 days and a high electrical conductivity of 1800 S/cm respectively^29,30^. Overall, GO served as effective barriers to ionic transport and abrasive-resistance enhanced additive in polymer coatings.

To further enhance the coating properties, PDMS was added as modifiers into the blended resins during the preparation of the anti-corrosion coatings^31^. In addition, a high bond energy that was possessed by the Si–O–Si- structure along with a large bond angle resulted the PDMS to have good thermal stability and elasticity, whereas, the hydrophobicity attributes and non-polar groups were associated with the –${\mathrm{CH}}_{3}$ groups, which resulted the PDMS to have a low surface energy and good hydrophobicity^31^. Hence, the incorporation of PDMS with organic resins (e.g., acrylic, epoxy, etc.) resulted in the improvement of thermal stability, physico-mechanical properties and anti-corrosive attributes which include good UV, processibility, durability, chemical and weather resistance, flexibility and toughness along with thermo-oxidative stability respectively^32^.

In the present work, GO-based polymer nanocomposite coatings with different inclusions of GO nanoparticles were fabricated along with the addition of PDMS. In terms of characterization, the thermal stability of the fabricated hybrid coatings was evaluated by the TGA analysis. Furthermore, via the utilization of UV–Vis spectroscopy and WCA instrument respectively, the degree of transparency and surface wettability of the coatings were investigated. The adhesion quality of the nanocomposite coatings to the substrate was evaluated by the CHT method. Finally, the surface morphology of the coatings that was examined by FESEM was also demonstrated.

## Material and methods

### Materials

Acrylic polyol resin, (A) (Product Name: Desmophen A 870 BA) and its curing agent aliphatic poly-isocyanate resin (NCO) were both purchased from Synthese, Malaysia and Bayer Material Science, Germany. Epoxy resin (EPIKOTE 828) and its curing agent cycloaliphatic amine (EPI-KURE 3388) were both obtained from ASA Chemicals, Malaysia. Hydroxyl-terminated PDMS (HT-PDMS) which was used as modifier was purchased from Sigma-Aldrich, Malaysia. Isophorone diamine (IPDA) was used as a cross-linker in the blended coatings. The hydrophobic graphene nanoplatelets in powder form (PCode: 806668-25G) composed of carbon (> 95 wt.%) and oxygen (< 2 wt.%) and bulk density of 0.04 g/ml was purchased from Sigma-Aldrich, Malaysia.

### Fabrication of GO/GO-based composite coatings

The GO was fabricated based on the method demonstrated by Sachin S.A. Kumar and his research group^6,15,29,30^. In this approach, functionalization, neutralization, filtration and drying were the four basic steps that was incorporated to fabricate the GO nanoparticles. Firstly, 1 g of graphene nanoparticles were transferred into a conical flask and in the ratio of 3:2:1, sulphuric acid, nitric acid, and potassium hydroxide were respectively added into the flask. At an ambient temperature, the mixture was stirred for 4 h at 550 rpm. Moreover, the filtration process was conducted using high quality filter paper. Due to the functionalization process, this resulted the graphene nanoparticles to become more acidic, therefore, the neutralization process (via washing) was conducted. In this process, the acidic solution was washed with deionized water under stirring for 40 min at 550 rpm. The washing was repeated until the pH ~ 7 was achieved. Subsequently, the dispersion was stirred continuously for 24 h to uniformly disperse the graphene nanoparticles and mainly to breakdown the larger size of graphene nanoparticles to smaller size in nanometer (nm) range. Then, under the influence of vacuum, the dispersion was then poured into a filtration unit. Here, it was vital to ensure that the filter paper containing the nanoparticles was completely dried before removing it from the filtration unit. Then, the final product, GO nanoparticles (in film form), was then peeled away gently from the filter paper and placed onto a petri dish followed by the drying process that was conducted in an oven for 12 h at approximately 80 °C.

In this study, a total of six samples were prepared by incorporating different loading rates of GO (in wt.%) into the polymer coatings. For instance, 0.5, 1, 2 and 3 wt.% GO were incorporated into the polymer matrix. On the other hand, two additional samples were prepared using the GO nanoparticles that were synthesized without the influence of vacuum (1 wt.% and 3 wt.%). Importantly, this step was taken in order to obtain a deep understanding on how the size of the GO nanoparticles affected the performance of the coating. These samples were then compared with the remaining samples fabricated with the influence of vacuum. To differentiate between these two samples (without vacuum) with the 1% and 3% GO (with vacuum), the corresponding codes were referred to as 1% GO (N) and 3% GO (N) respectively for the samples fabricated under the vacuum influence, as shown in Table 1.

**Table 1:** Compositions of all the prepared coating systems along with their corresponding designation.

Sample code	Acrylic resin (g)	Epoxy resin (g)	GO nanoparticles (g)	Presence of vacuum	Dry coating film thickness, µm (Avg.)
0.5% GO	9	1	0.05	Yes	123
1% GO	9	1	0.1	No	131
1% GO (N)	9	1	0.1	Yes	176
2% GO	9	1	0.2	Yes	278
3% GO	9	1	0.3	No	380
3% GO (N)	9	1	0.3	Yes	477

**Figure 1:**
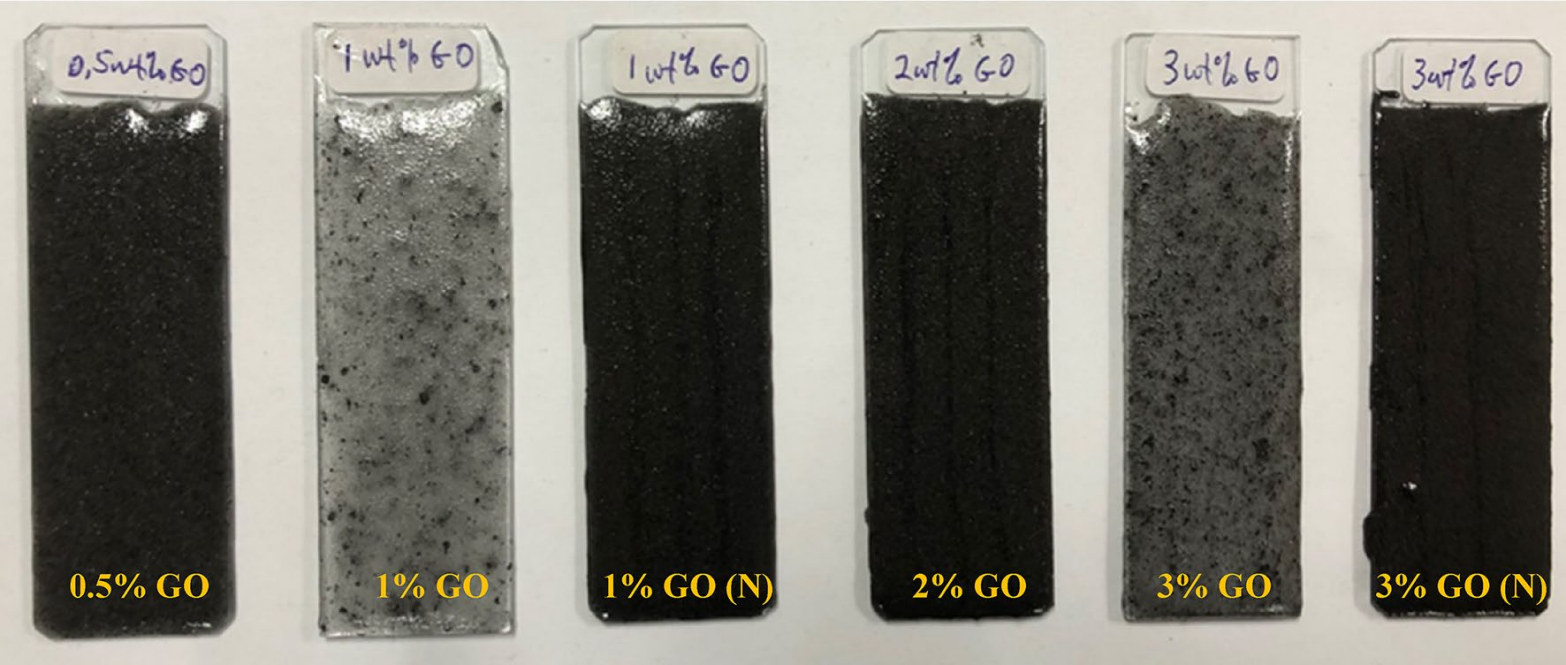
The coated microscope slides (glass) substrates for each corresponding sample (0.5–3% GO) respectively.

By using an acrylic to epoxy ratio of 90:10 (90A:10E), the resins were measured accordingly using a weight scale and transferred into a beaker. Different content ratios of freshly fabricated GO nanoparticles (0.5, 1, 2 and 3 wt.% respectively) were then added to the mixed resins in the beaker. The mixture was then stirred at 400 rpm for 15 min at room temperature. The sonication process was carried out for an additional 15 min followed by the addition of IPDA. Further sonication was done for 2 more minutes and then 1 wt.% of PDMS was added into the mixture. The mixture was then stirred again for 15 min. Subsequently, NCO curing agent was incorporated followed by stirring the mixture mechanically using a glass rod for 3 min. The final blended coating mixture was applied on both sides of the steel substrates and was left to dry for 5 days at ambient temperature. The steel substrates were abraded with a sandblaster (ASTM D609 standard) followed by acetone degreasing. Moreover, illustrated in Table 1 are the compositions of the prepared coating systems along with their corresponding designation and the average thickness of dry coating film after being applied on the substrate surface. Depicted in Fig. 1 are the coated substrates for each corresponding sample respectively. On an important note, among all the coating samples, a lighter colour was revealed by the 1 and 3 wt.% GO coating sample that was fabricated without the influence of vacuum. This clearly revealed that the resulted larger size of GO nanoparticles had dispersed unevenly within the polymeric matrix, hence leading to the phenomena of agglomeration. On the other hand, the coating samples that consisted the GO fabricated under the influence of vacuum possessed size in the nm range which dispersed uniformly within the polymeric matrix, thus, revealing a much darker coating texture. Hence, the colour contrast of these coating samples had demonstrated the importance and differences between the GO synthesized with and without the influence of vacuum respectively.

### Characterizations

By using Mettler Toledo TGA Q500 equipment (purchased from Perkin Elmer, Pyris Diamond, USA) that runs dynamically from 30 to 800 °C at a heating rate of 10 °C/min under nitrogen gas with a flow rate of 20 ml/min, the thermal stability of the hybrid nanocomposite coating samples was evaluated by the TGA tests. The UV–vis spectrometer (Shimadzu, Kyoto, Japan) in transmission mode at a wavelength range of 200–800 nm was utilized to analyse the transparency of the nanocomposite coatings coated on the glass substrates, as shown in Fig. 1. The main purpose of this analysis was to evaluate the percentage changes of the film transparency upon the addition of GO nanoparticles into the polymer matrix. The WCA test were carried out to evaluate the hydrophobic performance of the developed nanocomposite coatings by sessile drop method using OCA 15 EC instrument (Data Physics Instruments GmbH, Filderstadt, Germany). At different locations, 5 droplets of 5 µL distilled water was dispensed on the surface of the specimens and the images of static contact angles were respectively captured. An average of three readings were recorded the highest value was reported with less than 2° as a measurement error. The effect of the GO on the substrate/coating adhesion was analysed by the CHT test, following the ASTM D3359 B standard and the images were respectively captured. The surface morphology of the GO nanoparticles into the polymer matrix (free films) was closely examined by FESEM. All the free film nanocomposite samples were coated with platinum by using platinum sputter coater (Bio-Rad, Watford, England) in order to minimize the charging effects.

## Results and discussion

### Thermal analysis

**Figure 2:**
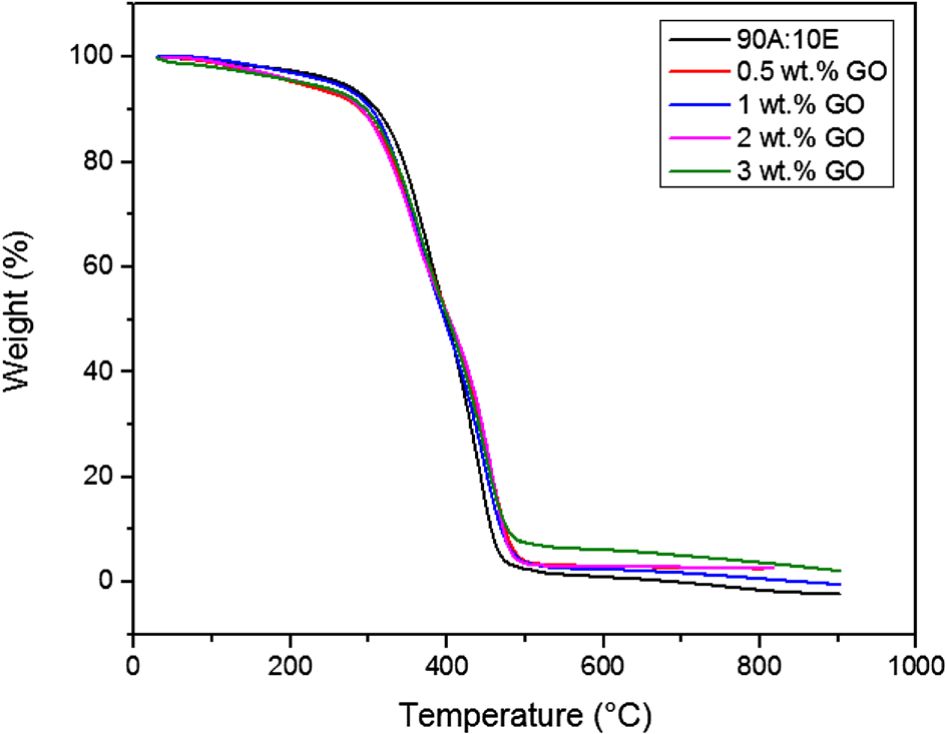
TGA curves of the acrylic-epoxy (90:10 wt.%) polymer coating along with the GO based nanocomposite coating samples that was fabricated under the influence of vacuum respectively.

In general, TGA is frequently employed to determine the decomposition of the polymers at various temperatures and the thermal stability of the materials respectively^33^. Defined as the temperature that corresponded to the weight equal to 5%, the initial degradation temperature (IDT) was employed as a thermal stability indicator for the developed coatings. Furthermore, Fig. 2 illustrates the TGA curves for the acrylic-epoxy (90:10 wt.%) polymer coating along with the GO based polymer nanocomposite coating samples for heating temperature ranging from 30 to 900 °C. Furthermore, roughly 1% of mass loss was observed at the 1st stage at 100 °C and below and this occurrence was due to the loss of moisture in the mixing of different ratios of the resin-based coating. In addition, in the 2nd stage, after 200 °C, the degradation and decomposition of the molecular networks of the acrylic-epoxy polymer coating was indicated by the TGA curve, whereby the thermal deterioration of the polymer coating had occurred in a single stage. Moreover, approximately between 280 and 450 °C, the curve indicated the genuine degradation zone of the polymer coating, which was due the breakdown of the linear carbon–carbon chains and the polymer’s crosslinked network respectively^34,35^. Additionally, it was observed that the evaporation of the moisture had resulted in the weight loss, which occurred between 80 and 100 °C. On the other hand, the carbonization of the residual polymer coating sample resulted in the weight loss at temperature exceeding 450 °C^36^. As mentioned previously, a one-step reduction that was exhibited by the acrylic-epoxy polymer coating confirmed its stability without further aggregation. It is also noteworthy to mention that at the point of inflexion from 220 to 300 °C, the smaller loss in acrylic resin percentage resulted the mass reduction to take place at a slower rate, however, the loss of mass was observed to occur at a faster rate at temperatures ranging between 320 and 450 °C respectively. Similarly, these findings were in complete agreement with the literatures which reported that the inclusion of epoxy resin had significantly reduced the degradation rate of the acrylic resin, whereas, the inclusion of large amount of mixture component into the epoxy network had resulted in the enhancement at the initial stage of the polymer decomposition^37^. In the recent years, PDMS has been reported to have good thermal and adhesion properties, however, its mechanical properties are comparatively weak. Furthermore, it was reported that in order to improve the mechanical properties of the PDMS, it was essential to blend the PDMS with various polymers and nanofillers^38^. Depending on the component amounts, it was previously demonstrated that the blended polymer exhibited various properties such as mechanical, thermal and chemical properties respectively^38^. The addition of PDMS with other resins has shown promising improvements in structural, morphological, thermal and mechanical properties. In this work, it is understood that the cross-linking network formation of the blends due to the functional groups available in acrylic and epoxy (C–O, C=O, OH) and PDMS (Si–O–Si) would play an important role in the enhancement of the thermal properties of the coatings developed. As depicted in Table 2, the acrylic-epoxy polymer coating had exhibited an IDT value of 293.23 °C. Moreover, the temperature where 50% weight loss occurred and the residue yields along with the IDT values for all the coatings were tabulated together, as shown in Table 2. A two-step decomposition had occurred for all the investigated coating samples. Here, it was clearly observed that the decomposition stages of the polymer matrix made up of acrylic and epoxy resins were not affected by the presence of PDMS and GO nanofillers respectively. Furthermore, the TGA curves suggested that the thermal stability of the polymer coatings was significantly enhanced with the addition of GO nanoparticles. In fact, due to the presence of the strong intermolecular force, the addition of GO nanoparticles into the polymer matrix increased the stability of the coating^39^. Firstly, from 0 to 310 °C, it was observed that the weight loss for all coating samples occurred due to the release of physiosorbed water molecules and the removal of the oxygen-containing functional groups which existed on the GO sheets^40^. This phenomenon further confirmed its hydrophilic nature. In addition, from the temperature region of 310–500 °C, the phenomena of the weight loss were due to the decomposition of the C–C bond skeleton of the GO^41,42^. Moreover, it was observed that all the coating samples did not exhibit any significant weight loss in the range of 500–900 °C. Interestingly, a residual yield in the range between 2 to 5% was obtained for all the GO coating samples at 700 °C, thus, indicating that a small part of the GO was burned into the volatile gases mostly at high temperature. Alternatively, Boiubed et al. investigated the thermal stability of neat GO coating composite sample^40^. Here, the TGA results for neat GO revealed three weight loss steps, whereby the weight loss occurred at 0–125 °C (1st step), 125–310 °C (2nd step) and 310–550 °C (3rd step) temperature regions respectively. Also, 16% of residual weight was observed for the neat GO sample at 700 °C, which revealed that most part of the GO was burned at high temperature. In other words, in this analysis, it can be clearly concluded that the chemical reaction of the blended resins and PDMS with the oxygen containing groups that existed on the GO surface enhanced their decomposition temperature and prevented the carbon skeleton from decomposition. Hence, GO inclusions into the polymer matrix is advantageous to enhance the thermal stability due to the equal dispersion and strong interfacial adhesion of the coatings, whereby it acted as a barrier to delay the decomposition of the blended resins.

**Table 2:** The TGA parameters for all the coating systems.

Coating system	Initial degradation temperature, IDT (ºC)	Temperature of 50% weight loss, ${T}_{50 \mathrm{wt}\%}$ (ºC)	Residue yield (%) at 700 ºC
Polymer coating (90:10 wt.%)	293.23	399.82	0.041
0.5 wt.% GO	285.95	401.76	2.742
1.0 wt.% GO	290.01	396.92	2.157
2.0 wt.% GO	290.20	400.21	2.888
3.0 wt.% GO	293.29	401.95	4.664

On the other hand, as depicted in Table 2, there was clear evidence that the recorded IDT values exhibited an increasing trend when more GO nanoparticles were employed within the hybrid polymer matrix. Similarly, when comparing 0.5% G and 1% G respectively, the temperature at 50% weight loss was observed to slightly decrease from 401.76 to 396.92 °C, and then it increased up to 401.95 °C as the GO content increased up to 3%, thus maintaining the temperature. Here, it can be concluded that thermal stability of the nanocomposite coatings had improved and did not have any significant degradation. Furthermore, it was previously reported by other researchers that agglomeration within the polymer matrix resulted in a decrease in the thermal stability of the coatings^43,44^. Despite experiencing agglomeration particularly for samples with higher GO loadings, the results indicated that these coatings did not exhibit a reduction in the thermal stability. Additionally, this exhibited results further indicated that the GO resulted in the enhancement of the physical barrier effect of the hybrid coatings (Fig. 2). To support this finding, Sharif et al. investigated on the thermal properties of the modified GO and PDMS nanocomposite coatings^45^. Here, it was revealed the inclusion of GO nanoparticles have enhanced the thermal and mechanical properties of the PDMS compared to using neat GO^45^. Moreover, as mentioned above, it was confirmed that the stronger interactions between the GO surface functional groups and the PDMS resulted in a lower mobility of the binder chains, thus, decreasing the rate of degradation of the samples which would have further enhanced the cross-linking density and hence resulting in the enhancement of the thermal properties^45^. In addition, the slow degradation of the polymer chains that was absorbed at the matrix filler interface could possibly result in the significant enhancement of the coating’s thermal stability. Interestingly, it was also revealed that the residual mass at 700 °C increased from 2.742 to 4.664% with the increment of the GO nanoparticles (0.5–3%) respectively. In short, the tortuous path effect provided by the presence of GO nanofillers within the polymer matrix resulted in the enhancement of the thermal stability of the coatings, hence delaying the escape of the volatile degradation products and the permeation of oxygen and char formation respectively. Similarly, these findings involving the incorporation of GO into other various types of polymers (e.g., polyurethane, epoxy etc.) were also found to be in accordance with the results reported by the researchers^13,39,40^.

### Degree of transparency

**Figure 3:**
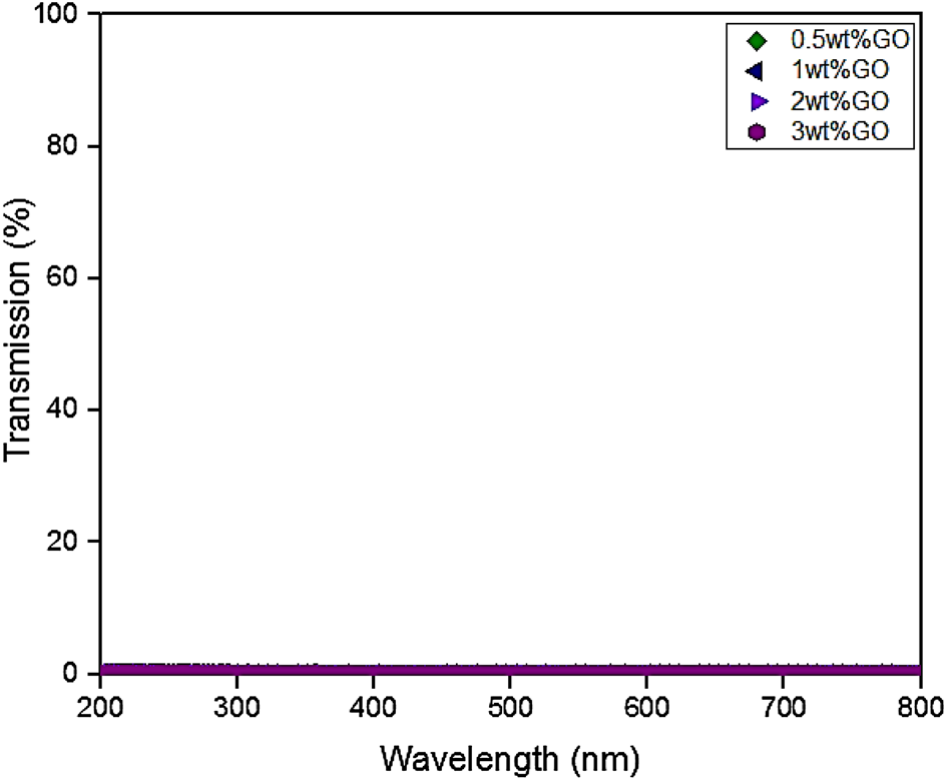
UV transparency curves for the GO based hybrid coating samples fabricated under the influence of vacuum.

UV–vis transmittance distribution is an essential property for each hybrid coating sample and this distribution was measured in the visible wavelength range of 200–800 nm, as depicted in Fig. 3. In addition, due to its extraordinary optical features, the graphene nanoparticles have the capability to tune the optical properties of the composite coating. In the past decade, it was reported that 2.3% of incident light was absorbed by a single layer of graphene in extremely broadband range^46^. In addition, the incorporation of this remarkable feature into graphene-based composite materials is still lacking in research as only several works have been previously published up to date. For instance, Wang et al. investigated the optical transmittance spectra by depositing GO sheets onto the polymer substrate^47^. Here, the graphene thin film, composed of GO sheets, with a thickness of 10 nm exhibited an optical transmittance of approximately 70%. For instance, graphene/GO film with a thickness of 10 nm has exhibited an optical transmittance of 70.7% at a wavelength of 1000 nm, whereas, as the film thickness decreases, this resulted the optical transmittance to significantly improve (> 80%)^47^. Moreover, Bao et al. demonstrated that by just employing 0.7 wt.% of graphene in pristine polymer, a tenfold rise of the optical absorption of a composite was observed in an ultraviolet-near infrared (UV-NIR) range^48^. In other words, the tunability of optical properties plays a vital role for progress in the application development, however, there is a lack of literature in relation to the optical transmission/absorption of graphene-based composite materials since it has only been investigated in visible or NIR range. Hence, significant efforts are required to systematically investigate the influence of GO content on these properties. In this approach, the optical properties of GO-based coatings with variable concentrations (0.5–3 wt.% GO) in PDMS polymeric matrix were investigated. Furthermore, it was clearly observed that the lowest content of GO (0.5 wt.%) was sufficient to totally block the incoming irradiation. By referring to Table 1 and Fig. 1 respectively, it can be seen that the coating thickness for all samples was reported to be high, in the range between 123 to 477 µm. Therefore, it was expected that optical transmittance to be extremely low, as illustrated in Fig. 3. Alternatively, Qi Wang et al. investigated the optical transmittance of the PDMS based microcrystalline graphite powder composites samples having different concentrations of graphite powder (0.15%, 0.25% and 0.42%) within the 300–1000 nm wavelength range^49^. The results were compared with sample containing pure PDMS. Here, it was observed that the pure PDMS sample exhibited 90% transmittance, however, the sample containing the highest content of graphite powder (0.42%) exhibited the lowest transmittance close to zero percent^49^. In other words, by incorporating higher content of graphene and GO nanoparticles, the thickness of the coating/film will increase, thus, resulting the optical transmittance to be extremely low (approaching zero percent). Furthermore, in another study, Zeranska-Chudek et al. reported similar trends at 0.5 wt.% graphene loading, whereby the drop of transmittance reached a saturation point, in this case zero percent transmittance, where no light was transmitted by this coating sample, thus, making it completely opaque in the range between 200 to 800 nm^50^. In addition, it was further demonstrated that the samples containing the lowest graphene content (< 0.02 wt.%) were almost fully transparent, however, as the content of graphene increased to 1.5 wt.%, the composite totally blocked the visible light^50^. Intuitively, the addition of graphene/GO nanoparticles into the polymer matrix lowered the transparency almost evenly, without changing the shape of the spectra. Hence, all the coating samples exhibited similar feature to single or multi-layered GO composite coating sample in the UV-IR range. In short, due to the relatively high thickness of the coating samples and with the increment of GO addition into the polymer matrix, this resulted the optical transmittance to be unmeasurable.

### WCA measurement

WCA measurements were carried out to investigate the hydrophobic attributes and the surface wettability of the fabricated coatings. Furthermore, it was revealed that acrylic resins had a good barrier property and were able to slow down the corrosion process with a CA of 74.3°^51^. Alternatively, Kocijan et al. demonstrated that the epoxy coating had exhibited a WCA of 75°^52^. However, it was also reported that a CA of approximately 65.1° was exhibited by an unfilled epoxy coating^53^. Here, it was indicated that both acrylic and epoxy coatings were hydrophilic in nature. Furthermore, to achieve an improved hydrophobic solid surface, Jiang et al. and Wang et al. demonstrated two main factors that are needed to be considered^54,55^. Firstly, the chemical composition played an essential role to enhance the surface hydrophobicity. For instance, the incorporation of the PDMS into the polymeric matrix further increased the CA of the coating. Secondly, the enhancement in hydrophobicity of the coating can be directly inferred with an increase in the CA after incorporating GO nanoparticles. Here, compared to micron size particles, the addition of graphene and graphene-based nanoparticles as nanofillers in the polymeric matrix greatly enhanced the surface roughness of the coating due to their higher specific surface area and smaller particle size^56^.

**Figure 4:**
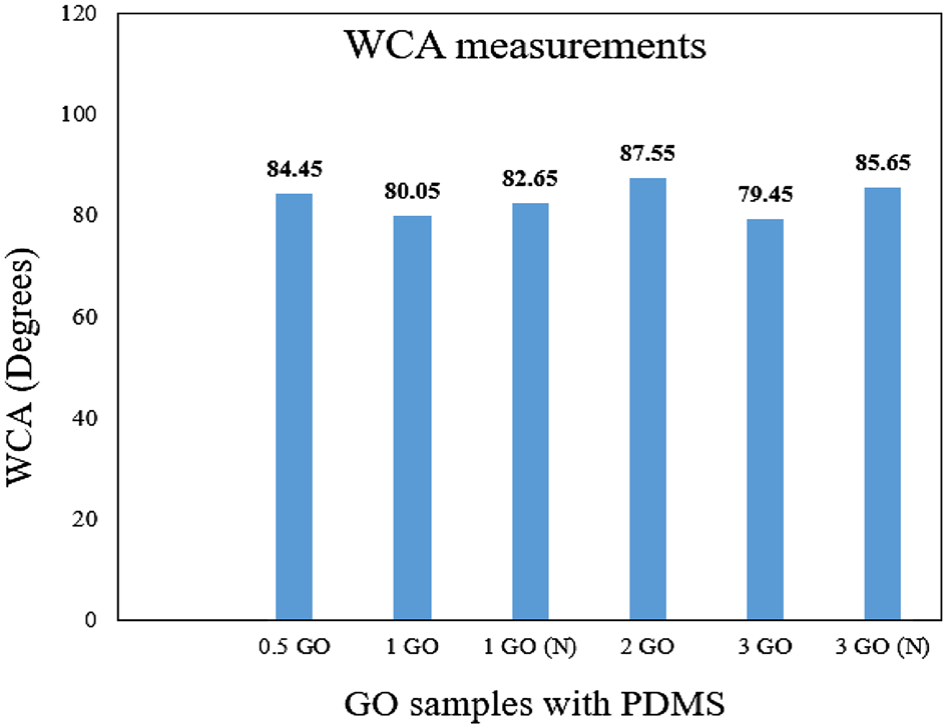
WCA values (degrees) for all the coating samples.

**Figure 5:**
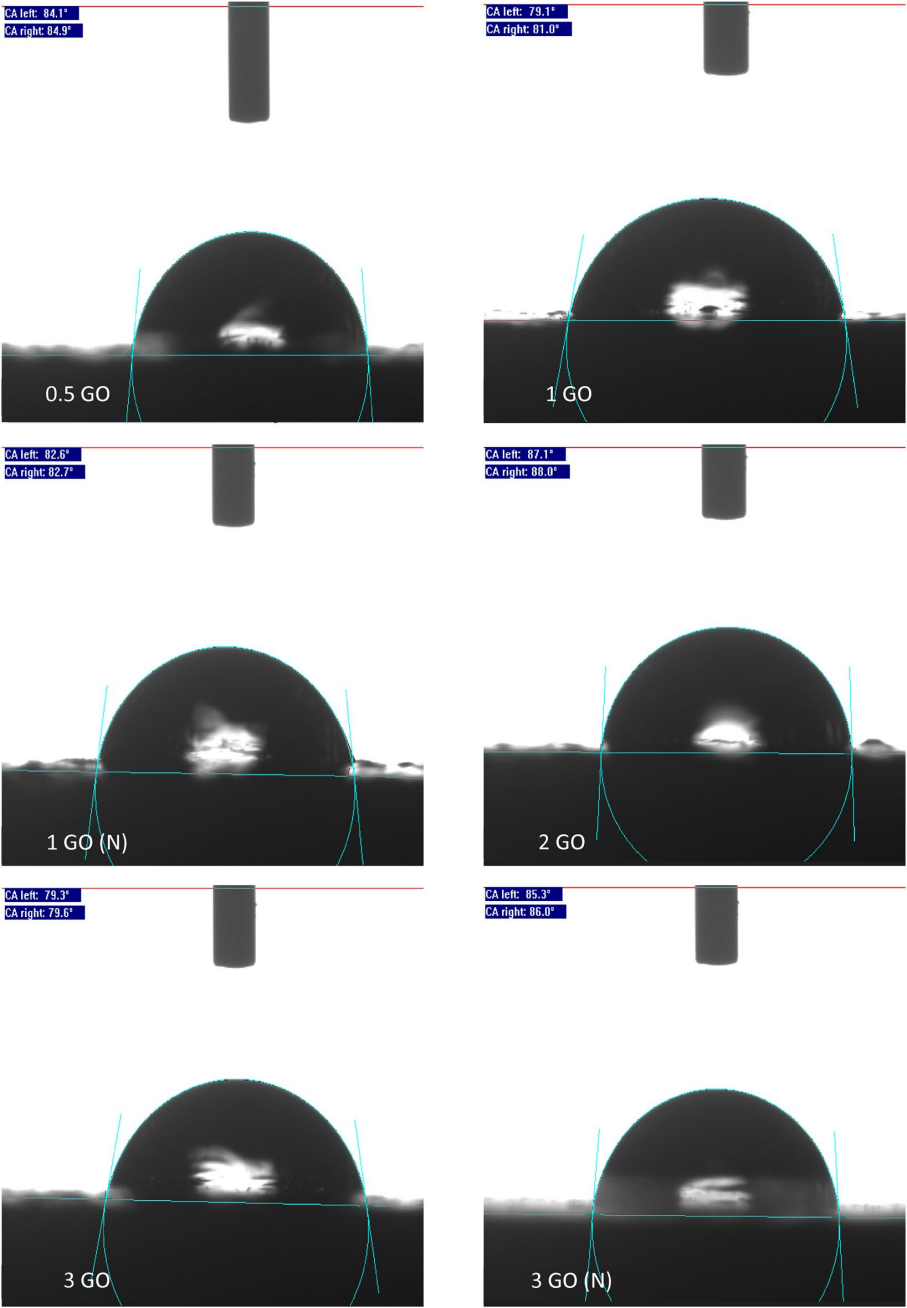
The representations of the WCA surface characterization for all the samples.

Therefore, in this evaluation, the WCA results for all the hybrid coatings containing PDMS and different GO content were included along with the addition of PDMS as a modifier of the resins to highlight the enhancement of the coating surface wettability towards greater hydrophobicity. Illustrated in Figs. 4 and 5 respectively are the recorded WCA values (degrees) and the obtained WCA surface characterizations for all the hybrid nanocomposite coating samples. In addition, it was observed that the GO inclusion into the polymer resins had enhanced the WCA up to 88°, close to hydrophobicity. On the other hand, as mentioned above, the surface wettability of the fabricated GOTF was previously investigated^6,29,30^. Here, it was reported that the GOTF exhibited a WCA of 50° and it did not degrade over 25 days, hence, exhibiting good resistance against UV degradation. To support these findings, it was reported that due to the presence of epoxy, carboxyl, and hydroxyl groups in the GO, invariably, the GO can possibly exhibit a CA up to 67.4°, thus, confirming the nature of hydrophilicity^15,57,58^. Alternatively, Xu et al. mentioned that the GO under typical treatment displayed hydrophilic properties, exhibiting a CA of approximately between 30° to 60°^59^.

Furthermore, it was observed that the GO fabricated without the influence of vacuum displayed a lower WCA, for instance, the 1% GO and 3% GO exhibited a WCA of 80.05° and 79.45° respectively. This was due to the presence of the large GO nanoparticles embedded within the polymeric matrix and resulted in agglomeration during the blending process. For instance, Ohigara et al. revealed that the hydrophobicity of the coating exhibited a downward trend particularly when the agglomeration of the nanoparticles increased^60^. Additionally, at higher concentrations, Chen et al. reported that the nanoparticles had the tendency agglomerate^61^. Here, it was demonstrated that for each coating system, there was a specific concentration of the GO that can be considered to be the optimum concentration, at which the surface morphology of the resulted surfaces can be significantly affected by the incorporated nanoparticles. In other words, exceeding this critical concentration resulted the GO to accumulate more at the bottom instead of the surface area of the coating film, thus, resulting the bulk properties to be affected more compared to the surface properties^61^. In this study, similar trends were observed when higher concentrations of GO were added (> 2 wt.%), which resulted the CA values to slightly decrease.

Also, Ogihara et al. stated that a low surface energy and high surface roughness resulted in a hydrophobic surface^60^. Here, it was also demonstrated that when a droplet of water was settled on a rough surface, the droplet was in contact with both the constituent materials and the air trapped within the rough surface. Hence, due to air being an absolutely hydrophobic material, this resulted to an enhancement in the surface hydrophobicity^60^. Here, compared to the other GO samples that was fabricated without the presence of vacuum, the 0.5%, 1%, 2% and 3% GO samples revealed a higher WCA of approximately 84.45°, 82.65°, 87.55° and 85.65° respectively. The optimum enhancement was exhibited by the 2% GO sample which exhibited the highest WCA value of 87.55°. In short, the addition of GO into the polymer matrix had remarkably improved the surface hydrophobicity, whereby GO loadings up to 2% was appropriate due to the optimum dispersion.

### Coating surface adhesion analysis

**Figure 6:**
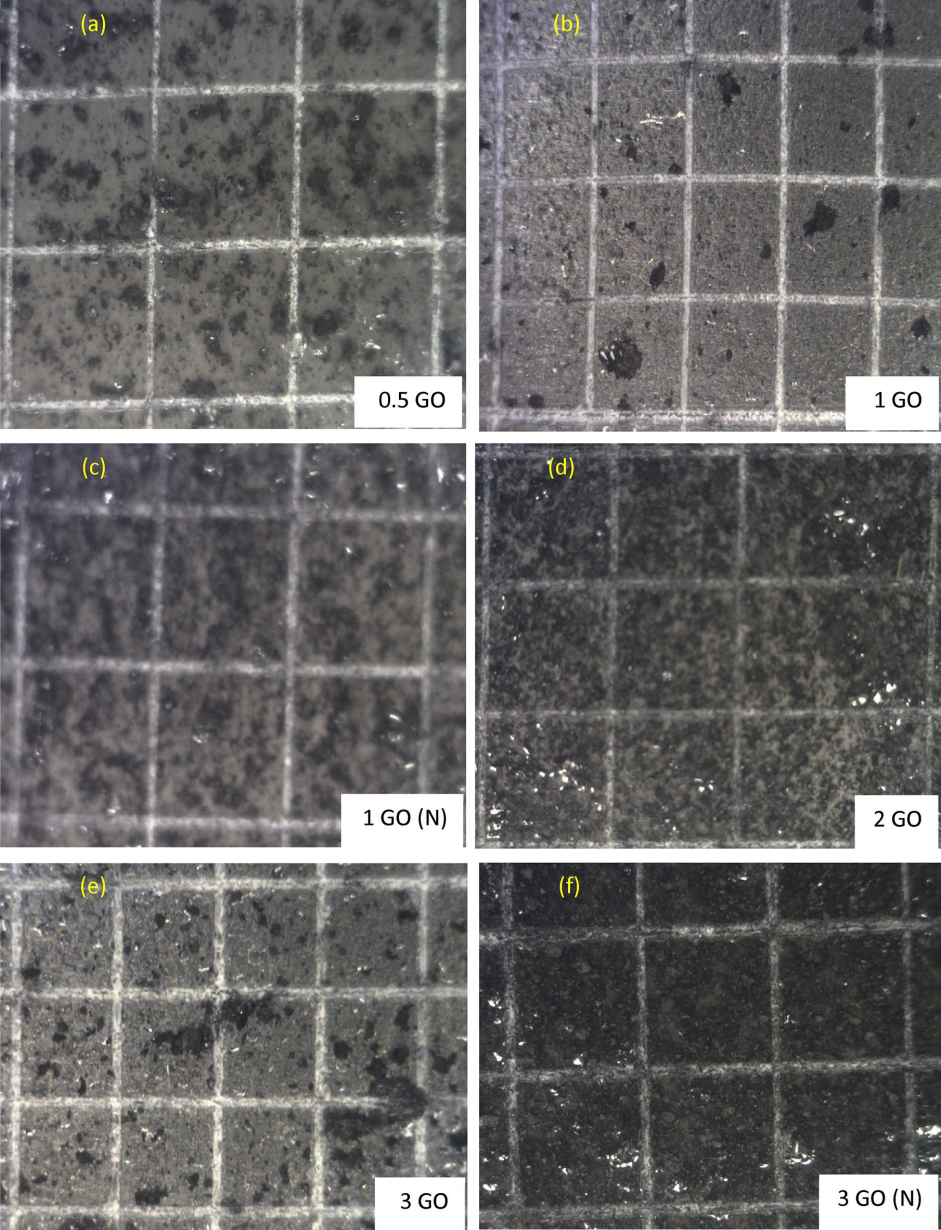
Representations of the CHT analysis performed on (**a**) 0.5 wt.% GO, (**b**) 1 wt.% GO, (**c**) 1 wt.% GO (N), (**d**) 2 wt.% GO, (**e**) 3 wt.% GO and (**f**) 3 wt.% GO (N) based nanocomposite coating samples.

When developing anti-corrosion coating samples, another essential property that mandatory to be analysed was the coating layer adhesion to the pre-treated substrate surface^62,63^. Moreover, shown in Fig. 6a–f are the adhesion behaviours of all the GO-based nanocomposite coating samples on the steel substrate. Figure 6a and b illustrated the surface adhesion image of 0.5% GO and 1% GO coating system respectively which exhibited no loss of adhesion, whereby all the edges of the cuts were smooth, which indicated excellent adhesion of the blended resins to the pre-treated steel substrate. Therefore, these coating systems were classified as 5B grade. Furthermore, similar observation which further confirmed that the inclusion of 1wt.% of GO nanoparticles into the polymeric matrix exhibited no effects on the superior adhesion properties of the blended polymer resins. Here, the surface of the 1% GO (N) and 2% GO sample was also rated as 5B grade, as illustrated in Fig. 6c. However, as illustrated in Fig. 6e–f, the 3% GO and 3% GO (N) coating samples were classified as 4B respectively due to the presence of the detached flakes at the intersections of the cut (< 5%). In short, the addition of GO into the polymer coatings exhibited no significant effect on the coating/substate adhesion whereby no peelings were observed in all coating systems^53^. The coating adhesion can be possibly decreased due to the presence of water at the metal/coating interface, thus, resulting the corrosion rate to accelerate on the metal substrate^64^. In other words, poor adhesion permits the corrosive ions to accumulate at the coating/metal interface, thus, triggering the degradation process to occur. Therefore, this surface adhesion analysis highlighted that the interphase adhesion of the coating was significantly improved with the incorporation of higher acrylic resin (host binder) compared to epoxy coating due to its good flowability. In short, it has been revealed that all the GO-based nanocomposite coating systems exhibited excellent adhesion behaviour of the coatings, receiving 5B and 4B ratings according to the ASTM standard.

### Surface morphology (FESEM)

**Figure 7:**
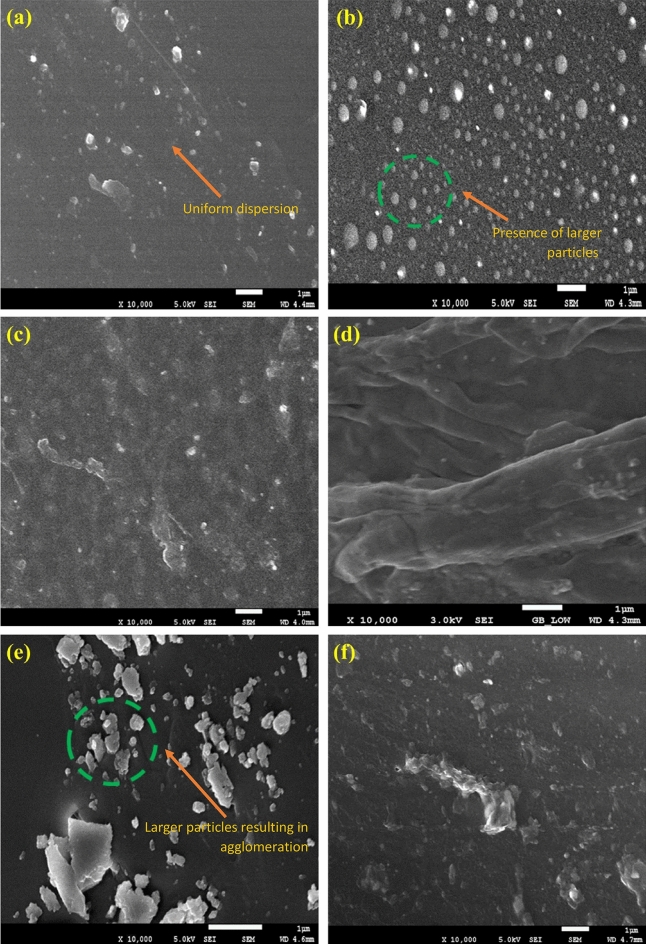
FESEM representations of the fracture surface of (**a**) 0.5 wt.% GO, (**b**) 1 wt.% GO, (**c**) 1 wt.% GO (N), (**d**) 2 wt.% GO, (**e**) 3 wt.% GO and (**f**) 3 wt.% GO (N) based hybrid polymer nanocomposites coatings respectively.

**Figure 8:**
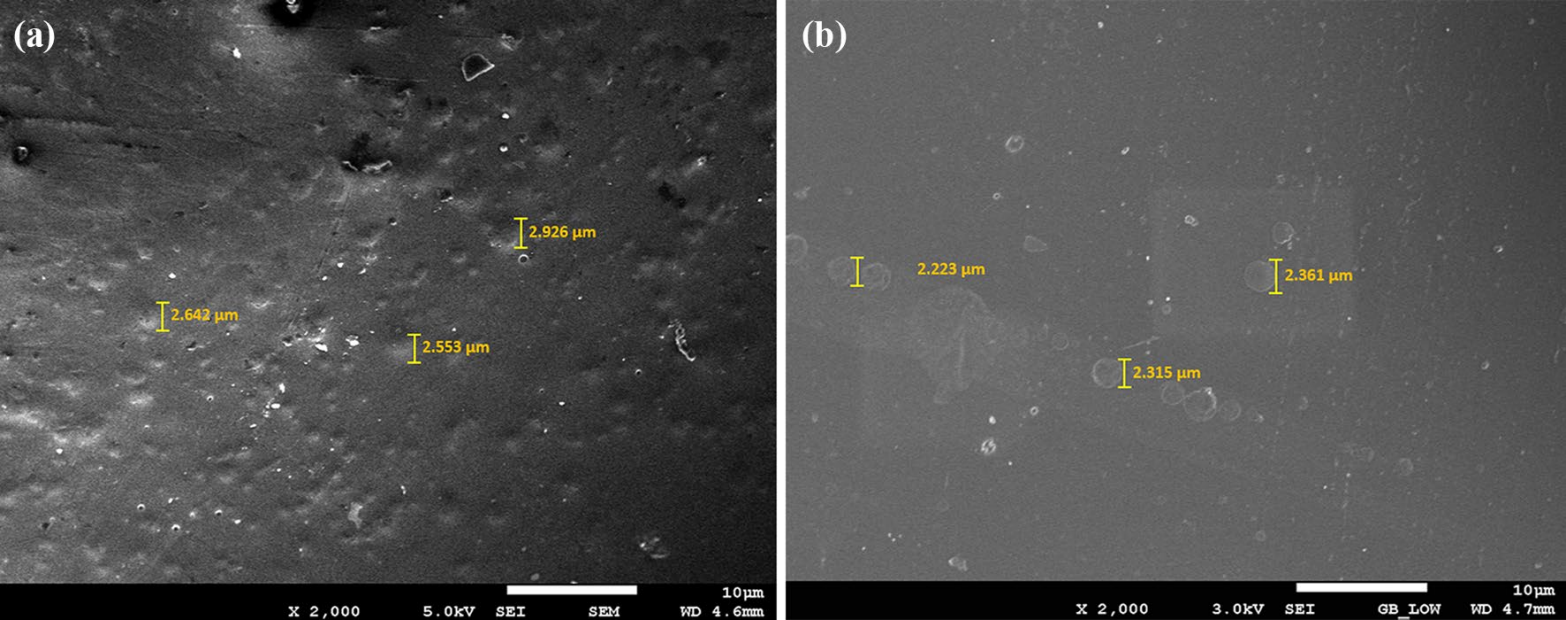
FESEM micrographs distinguishes the lateral dimensions of (**a**) 3 wt.% GO and (**b**) 3 wt.% GO (N) respectively.

The employment of different GO concentrations into the polymer matrix was conducted via the solution intercalation method. Based on this concept, the surface morphology of the resulted coatings had differed, whereby the incorporation of a higher amount of GO resulted to agglomeration. Hence, the dispersion of the nanoparticles in the polymeric matrix were affected^65,66^. In this approach, the FESEM was employed to clarify the surface morphology differences between the GO nanoparticles and the blended polymer resins. Illustrated in Fig. 7 are the micrographs of the prepared hybrid GO-based nanocomposite coating samples. Figure 7a–f represented the GO samples that were fabricated with and without the influence of vacuum respectively. Furthermore, as depicted in Fig. 7a, c and d respectively, there was clear evidence that the uniform dispersion of GO nanoparticles in the polymeric matrix had occurred, thus, indicating the efficiency of the sonication process to fabricate an acceptable dispersion level of GO-based nanocomposite coatings. On the other hand, it was observed that the agglomeration had increased with the increment of the GO nanoparticles, which was due to the increase in the number of nanoparticles in the unit area. The most noticeable effect was observed with the highest loading ratio (3%), as shown in Fig. 7f. Moreover, Fig. 7b and e further proved that larger GO nanoparticles were present within the polymeric matrix (highlighted in green). In order words, this phenomenon had increased the ability of the nanoparticles to attract each other, thus, resulting in the formation of relatively larger agglomerated nanoparticles. Similar findings on the agglomeration of nanoparticles with higher loadings were previously reported in these literatures which resulted the coating surface to have a hydrophilic attribute^6,40,61,67^. Last but not the least, the lateral dimensions of the highest loading rates (3 wt.%) of GO nanoparticles will be discussed and compared between the GO fabrication with and without the influence of vacuum. Figure 8a and b illustrates the lateral dimensions of the 3 wt.% GO and 3 wt.% GO (N) coating sample respectively. In addition, it was previously reported that the GO nanoparticles generally exhibited a diameter of approximately 1.2 nm^68^. Moreover, depending on the synthesis and post synthesis processes (e.g., sonication etc.), the lateral size of the GO nanoparticles can vary between tens to hundreds of micrometers (µm). Previously, Yang et al. investigated the dimensions of the GO nanoparticles via atomic force microscopy (AFM)^69^. Here, it was observed that the GO nanoparticles exhibited a thickness of approximately 1.1 nm and a lateral size ranging from 500 to 50 µm^69^. Recently, Rhazouani et al. reported that the AFM profiling showed that the GO nanoparticles had thickness of 1.5–2.5 nm and lateral dimensions of 1–1.5 µm respectively^68^. From Fig. 8a, the lateral dimensions of the 3 wt.% GO nanoparticles were observed to range between 2.553 and 2.926 µm, while, Fig. 8b illustrated that the 3 wt.% GO (N) nanoparticles exhibited a much lower lateral dimensions, ranging between 2.223 and 2.361 µm respectively. The differences in the lateral dimensions clearly revealed the significance of the influence of vacuum during the GO synthesis process. In other words, the 3 wt.% GO (N) sample that was fabricated under the influence of vacuum had resulted in smaller nanoparticle size. It is also noteworthy to mention that a lower loading rates of GO nanoparticles (0.5–2 wt.%), the lateral dimensions of the GO nanoparticles is expected to be much lower compared to the 3 wt.% GO sample. Hence, the results exhibited in Fig. 8a and b were in complete agreement with the previous reported literatures as well as the results reported in this investigation^68,69^. Therefore, it was confirmed that higher loading rates of GO nanoparticles and the fabrication of GO without the influence of vacuum had resulted in larger size nanoparticles, thus leading to agglomeration and lowering the corrosion protection performance. Overall, 0.5% GO, 1% GO and 2% GO revealed the best surface morphology in terms of being evenly dispersed/distributed, no cracks or holes, no obvious agglomeration, thus, confirming the good adhesion properties of the coating.

## Conclusion

In this present study, using different concentrations, the GO-based nanocomposite coatings were developed via the intercalation method. The thermal properties, optical transmittance/degree of transparency, surface wettability, surface adhesion and morphology of the coatings were investigated. It was reported that the GO nanoparticles within the polymeric matrix remarkably enhanced the anti-corrosion characteristics and the hydrophobicity of the fabricated coatings. Moreover, at the highest loading (3 wt.%), the addition of GO nanoparticles into the polymeric matrix resulted the WCA of the coating to decrease due to the agglomeration of the nanoparticles. Alternatively, 0.5, 1 and 2 wt.% GO coating samples which were fabricated under the influence of vacuum exhibited higher WCA values, whereby the 2 wt.% GO sample had the most pronounced effect of hydrophobicity, with a WCA of 87.55°. Here, it was concluded that the low surface energy of PDMS resin resulted in the alteration of the wettability of the nanocomposite coatings towards a more hydrophobic behaviour. In addition, according to the ASTM standard, all the nanocomposite coatings exhibited excellent adhesion behaviour of the coatings, receiving 5B and 4B ratings respectively. Finally, the combined incorporation of GO nanoparticles and PDMS into the blended polymer resins provided a tortuous path effect which greatly enhanced the thermal stability of the coatings, thus, delaying the escape of the volatile degradation products and the permeation of oxygen and char formation respectively. Overall, based on our experimental findings and analysis, we can conclude that the GO-based nanocomposite coatings serve as a better corrosion inhibitor, thus, providing an effective barrier to inhibit the rate of corrosion.

## Data Availability

The datasets generated during and/or analysed during the current study are available from the corresponding author on reasonable request.
